# Metabolic adaptation of a methanogen to a ferruginous environment associated with intracellular Fe- and S-rich nanoparticles

**DOI:** 10.3389/fmicb.2026.1967823

**Published:** 2026-10-07

**Authors:** Yidi Zhang, Donglei Zhang, Yizhi Sheng, Hongyu Chen, Gaoyuan Li, Hailiang Dong

**Affiliations:** 1 Center for Geomicrobiology and Biogeochemistry Research, State Key Laboratory of Biogeology and Environmental Geology, China University of Geosciences, Beijing, China; 2 School of Water Resources and Environment, China University of Geosciences, Beijing, China; 3 Frontiers Science Center for Deep-Time Digital Earth, China University of Geosciences, Beijing, China; 4 School of Earth Sciences and Resources, China University of Geosciences, Beijing, China

**Keywords:** ferruginous ocean, mackinawite, methanogenesis, proteomics, TEM-EDS, transcriptomics

## Abstract

Across the great oxidation event (GOE) period, the oceans experienced a major transition from ferruginous (Fe^2+^-rich) to localized euxinic (HS^−^-rich) conditions. In ferruginous oceans, sulfur is mostly sequestered into metastable aqueous Fe-S clusters (FeS_aq_) and sulfide minerals like mackinawite (FeS_Mack_). Similarly, in euxinic oceans, iron is sequestered in such clusters and/or sulfide minerals. However, it is unclear how ancient methanogens, with high demands for sulfur and iron, adapted to this critical transition. We hypothesize that FeS_aq_ and FeS_Mack_ could serve as sources of sulfur and iron to support cellular growth and methanogenesis. A model ancient methanogen *Methanococcus maripaludis* S2 (MmS2) was inoculated in a growth medium under either Fe^2+^-rich or HS^−^-rich condition. Biological utilization of FeS_Mack_ was imaged using transmission electron microscopy (TEM) and nanoscale secondary ion mass spectrometry (NanoSIMS). Transcriptomic and proteomic analyses were performed to characterize the cellular and biochemical responses of MmS2 under contrasting Fe-S conditions. The results revealed that MmS2 maintained a consistent methanogenic activity and growth irrespective of different Fe-S conditions. Under high-Fe^2+^ but limited HS^−^ conditions, Fe- and S-rich particles accumulated inside cells, indicating utilization of FeS_aq_ and/or FeS_Mack_ as sulfur sources. Intracellular Fe- and S-rich particles accumulation was accompanied by distinct transcriptomic and proteomic responses involving cellular metabolism and energy conservation. These findings provide new insights into the adaptive mechanisms of methanogens in response to evolving iron and sulfur environments during early earth, shedding light on the co-evolution of life and environment over geological history.

## 1 Introduction

The Hadean or Archean origin of methanogens has been accepted through multiple efforts, such as carbon isotopic records (Ueno et al., 2006), molecular clock dating (Wang et al., 2021), and geochemical variations of nickel availability (Konhauser et al., 2009). During this period and afterwards (>3.8–2.4 Ga), large amounts of Fe^2+^ were released into the oceans via hydrothermal fluids, sedimentary diagenesis, and anoxic weathering of continental rocks (Poulton and Canfield, 2011), resulting in Fe^2+^-rich yet sulfide-poor conditions known as ferruginous ocean conditions (Poulton and Canfield, 2011). Thus, ancient methanogens likely emerged and evolved in Fe^2+^-rich environments. It was not until ~2.4 billion years ago, during the Great Oxidation Event (GOE), that oxidative weathering of continental sulfide minerals increased the flux of SO_4_^2−^ into the oceans. Increased flux of SO_4_^2−^ enhanced the activity of sulfate-reducing bacteria to release H_2_S (Fakhraee et al., 2025; Planavsky et al., 2012). Although reactive sulfides could be buried as sulfide minerals (Planavsky et al., 2012), they can accumulate in certain marine regions (Poulton and Canfield, 2011), leading to the localized sulfidic (or euxinic) ocean (Canfield, 1998; Reinhard et al., 2009). The anoxic HS^−^-rich environment would have provided abundant HS^−^ sources to methanogens without causing biological toxicity (Shakeri Yekta et al., 2023). In response to such environmental transition from ferruginous to sulfidic condition, methanogens may have evolved to require more sulfur than iron, consistent with the typical composition of growth media used to cultivate methanogens, where the concentration of HS^−^ is typically 20–30 times higher than that of Fe^2+^ (Whitman et al., 1986).

Fe and S are essential to methanogenesis enzymes in the form of Fe-S clusters (Glass and Orphan, 2012). They have been shown to contain more Fe-S clusters than other organisms (Andreini et al., 2017). For example, *Methanococcus maripaludis* contains approximately 15 times more Fe-S clusters per milligram of protein than *E. coli* (Liu et al., 2010). Methanogens exhibit the highest abundance of putative Fe-S cluster-binding proteins among prokaryotes, with an average normalized gene abundance of 22.2 4Fe-4S motif-containing open reading frames (ORFs) per 1,000 total ORFs, far exceeding the values observed in obligate anaerobes *Desulfitobacterium hafniense* (~16.0), facultative anaerobe *E. coli K-12* (~9.1), and aerobic archaeon *Sulfolobus solfataricus* (~6.4) (Major et al., 2004).

In paleo-oceans, Fe and S were not always in the free forms for biological assimilation and synthesis of Fe-S clusters (i.e., Fe^2+^ and HS^−^). Excess Fe^2+^ in ferruginous oceans would react with aqueous HS^−^ to precipitate sulfide minerals. Likewise, high HS^−^ concentration in localized euxinic oceans would remove aqueous Fe^2+^ through sulfide mineral precipitation (Rickard and Luther, 2007; Saito et al., 2003). In both scenarios, metastable aqueous Fe-S clusters (FeS_aq_) are initially formed, which can subsequently condense into nanocrystalline Fe-S minerals, such as mackinawite (FeS_Mack_) (Luther and Rickard, 2005; Rickard, 2006; Rickard and Luther, 2007). Over time, FeS_aq_ or FeS_Mack_ may transform into crystalline greigite, pyrrhotite, and eventually pyrite or other sulfide minerals (Hunger and Benning, 2007; Luther, 1991). Therefore, in the specialized sulfate–methane transition zone (Sela-Adler et al., 2017), methanogens likely encountered FeS_Mack_ or iron sulfide minerals, not necessarily free Fe^2+^ or HS^−^ ions.

Interestingly, studies have investigated the potential role of FeS_Mack_ in early life processes due to its structural similarities to biological FeS clusters (Zhao et al., 2020). Theoretical models and experimental studies suggest that FeS_Mack_ can catalyze carbon fixation in terrestrial hot springs (Nan et al., 2024), and support C-N bond cleavage of cyanide under ambient conditions (Grosch et al., 2022). Previous studies have shown that microorganisms, including heterotrophic bacteria, can obtain Fe and S from Fe-S minerals (Boyd and Payne, 2025). The hyperthermophilic archaeon *Pyrococcus furiosus* can also assimilate FeS_aq_ clusters released from mackinawite as an Fe source (Clarkson et al., 2021). Moreover, Fez-dependent ferrosomes provide a further mechanism for intracellular Fe storage (Grant et al., 2022). However, whether FeS_Mack_ can serve as both an Fe and S source to sustain methanogen growth and methanogenesis remains unclear. Recent studies have shown that pyrite could be reduced by methanogen to produce FeS_aq_, which was speculated to be a source of Fe and S to support cell growth (Payne et al., 2023; Spietz et al., 2022a; Spietz et al., 2022b). Pyrite-grown *Methanococcus voltae* accumulated thioferrate-like nanoparticles and upregulated proteins associated with Fe acquisition and storage (Payne et al., 2021a), while mineral versus aqueous Fe/S sources remodeled the proteome and metalloproteome of *Methanosarcina barkeri* (Larson et al., 2024). In contrast, FeS_Mack_, as a more reactive Fe-S phase, may influence Fe and S availability through distinct dissolution and precipitation dynamics. However, these studies did not specifically examine whether methanogens could obtain limiting S or Fe from FeS_Mack_ under Fe^2+^-rich, HS^−^-limited or HS^−^-rich, Fe^2+^-limited conditions to sustain growth, methanogenesis, and other essential biological processes.

We hypothesize that FeS_Mack_ could serve as a source of Fe and S for methanogens in Fe^2+^-rich and HS^−^-rich environments, supporting their growth and metabolism under both ferruginous and euxinic conditions. To test this hypothesis, we selected *Methanococcus maripaludis* S2 (MmS2) and inoculated it in a medium containing synthetic FeS_Mack_. MmS2 has a fully characterized genome (Funkner et al., 2024; Richards et al., 2016) and grows rapidly (Goyal et al., 2016). It has a high requirement for Fe and S (Major et al., 2004) and harbors a methanogenic system potentially reflective of the metabolism of the last universal common ancestor (LUCA) (Weiss et al., 2016). By adding either excess aqueous Fe^2+^ or excess aqueous HS^−^ to FeS_Mack_, we simulated either Fe^2+^-rich or HS^−^-rich environment and minimized FeS_Mack_ dissolution. We then performed biological experiments to measure cell growth and methane production. Transmission electron microscopy coupled with energy-dispersive X-ray spectroscopy (TEM-EDS) and nanoscale secondary ion mass spectrometry (NanoSIMS) were employed to demonstrate bioavailability of FeS_Mack_ to MmS2. Finally, protein synthesis and gene expression were investigated for MmS2 in response to either Fe^2+^-rich or HS^−^-rich conditions. Here, we show that MmS2 acquired Fe and S from FeS_Mack_ and maintained growth and methane production under both conditions. We predicted that contrasting Fe and S availability would result in distinct patterns of intracellular Fe and S accumulation and associated molecular responses. Consistent with this prediction, Fe- and S-rich particles accumulated more extensively under Fe^2+^-rich conditions, accompanied by changes in genes and proteins involved in sulfur utilization and metal homeostasis. These findings provide insights into the physiological responses of a modern methanogen to contrasting Fe and S regimes relevant to ferruginous and euxinic environments over geological time.

## 2 Materials and methods

### 2.1 Mackinawite synthesis and characterization

FeS_Mack_ was synthesized according to a previous method (Butler and Hayes, 1998). Basically, aqueous Fe^2+^ reacts with HS^−^ to form amorphous FeS (Berner, 1967). When amorphous FeS aggregates into clusters larger than 150 unit cells, it precipitates as nanometer-sized mackinawite (FeS_Mack_) (Rickard, 2006). Briefly, 168 mL of 1.1 M HS^−^ (Na_2_S) solution was slowly added to 280 mL of 0.57 M Fe^2+^ (FeCl_2_) solution in a 500 mL anaerobic bottle. The reaction mixture was stirred for 3 days, after which it was transferred to a 50 mL centrifuge tube and centrifuged at 8,000 g for 10 min. The solid was resuspended in O_2_-free ultrapure H_2_O for 12 h and then stirred again. This process was repeated for 3–5 days, with a total of 8 equilibration cycles. The synthesized FeS_Mack_ was stored under anoxic condition until use. The synthetic FeS_Mack_ was characterized using X-ray powder diffraction (XRD) and TEM-EDS.

### 2.2 Mackinawite stability experiments

Because subsequent biological experiments were conducted under excess Fe^2+^ or excess HS^−^ conditions in a microbial growth medium (Supplementary Material 1.1), it was important to evaluate the stability of FeS_Mack_ under these conditions. Stability experiments were performed in the base salt medium in 50 mL serum bottles (20 mL suspension volume) using gradients of FeS_Mack_ (4.40–26.37 mg/L, or expressed as 50–300 μM to facilitate comparison across different treatments), aqueous Fe^2+^ concentration (50–300 μM), and HS^−^ concentration (50–300 μM). These Fe^2+^ and HS^−^ concentrations were based on sedimentary records and models of paleo-ocean chemistry (Holland, 1973). All bottles were thoroughly mixed, sealed with blue butyl rubber stoppers and aluminum caps, and then equilibrated at 37 °C for 4 h. Preliminary experiments demonstrated that this time was sufficient to reach equilibrium. After the reaction, the supernatant was filtered through a 0.22 μm membrane filter, which was prewashed using ultrapure H_2_O. The total Fe^2+^ concentration was quantified using the Ferrozine method (Viollier et al., 2000), while the total HS^−^ concentration was measured via the methylene blue method (Strocchi et al., 1992). The detection limits for Fe^2+^ and HS^−^ were 5 μM and 1 μM, respectively. TEM was used to characterize the reacted FeS_Mack_. Aqueous geochemical modeling software Visual MINTEQ (Version 3.1) (Gustafsson, 2013) was used to calculate mineral dissolution or precipitation under these conditions.

### 2.3 Growth experiments of *Methanococcus maripaludis* S2

The Fe- and S-free culture medium was initially prepared by dispensing 25 mL of the O_2_-free base salt medium to 50 mL culture bottles, which were sealed with blue butyl rubber stoppers and aluminum caps, and subsequently autoclaved at 121 °C. After cooling to room temperature, vitamin stock and trace element stock were added (Supplementary Material 1.1). Cells were cultured under non-N_2_-fixing conditions with 5 g/L NH_4_Cl as the nitrogen source and 27 g/L HCOONa as erve as the methanogenic substrate. Subsequently, a set of representative FeS_Mack_-Fe^2+^-HS^−^ combinations, i.e., either 50 μM FeS_Mack_ alone or in combination with 50, 150, 250 μM of either Fe^2+^ or HS^−^, were added to the bottles and equilibrated at 37 °C for 4 h. Finally, MmS2 culture, pre-grown in the base salt medium to deplete background Fe and S from chemical reagents, was inoculated into the bottles. Triplicate experimental bottles were incubated in the dark at 37 °C.

Subsamples were taken from the bottles every 24 h for time-course measurements of methane production, cell growth, and aqueous concentrations of Fe^2+^ and HS^−^. For methane concentration, a 100 μL subsample of headspace gas was drawn with a sterile, N_2_-flushed syringe, injected into a gas chromatograph (Agilent 7890A, California, United States) equipped with an HP-5MS column (Agilent 19091S-433UI, California, United States) at 100 °C, and measured with a flame ionization detector (FID) at 250 °C, using N_2_ as the carrier gas. A calibration curve was established using CH_4_ and N_2_ (purity > 99.99%) as standards. The instrument had a detection limit of <0.1% (CH_4_ in N_2_, v/v).

Due to the difficulty of cell enumeration or optical density measurement in the presence of FeS_Mack_, the growth of *Methanococcus maripaludis* S2 was monitored by measuring time-course change of DNA concentration. Total DNA has been used as a proxy for biomass because DNA content is correlated with cell number (Gong et al., 2021). However, previous studies have shown that culture conditions can influence cell size and cell density in MmS2, potentially affecting DNA-based biomass estimates (Payne et al., 2023). Therefore, DNA concentration in this study should be interpreted as an indicator of relative biomass changes rather than absolute cell numbers. Prior to sampling, the experimental tubes were thoroughly shaken to resuspend the cells. One milliliter suspension was taken using a N_2_-flushed sterile syringe and transferred to a 2 mL centrifuge tube. After centrifugation at 10,000 g for 15 min, 900 μL of the resulting supernatant was discarded. Next, the cell pellet was lysed and the released DNA was purified as described previously (MP Biomedicals, Irvine, CA) (Spietz et al., 2023). The amount of DNA was quantified using a Qubit HS dsDNA kit and fluorimeter (Invitrogen, Massachusetts, United States).

Over the same time frame, 1 mL cell suspension was anaerobically extracted from the experimental bottles and passed through a 0.22 μm sterilized and washed filter. The filtrate was used for measurements of Fe^2+^ and HS^−^ concentrations as described above.

### 2.4 Determination of intracellular iron and sulfide contents

Logarithmic-phase cells in 50 mL culture were harvested under strictly anaerobic conditions by centrifugation at 4000 g for 10 min at 4 °C. To remove extracellularly adsorbed metals, the cell pellet was resuspended in 20 mL sterile and anaerobic base salt medium containing 5 mM nitriloacetic acid as a complexing reagent, mixed thoroughly for 10 min by gentle inversion, and centrifuged again at 4000 g for 10 min at 4 °C. This rinsing process was repeated three times. The washed cells were resuspended in 2 mL anoxic ultrapure water and disrupted via ultrasonication. The lysed cells were centrifuged at 8000 g for 10 min, and the supernatant was collected. An 0.1 mL aliquot of the supernatant was used to determine protein concentration using a Qubit protein assay kit and fluorometer (Invitrogen, Massachusetts, United States). The remaining supernatant was divided equally into two fractions. One fraction was measured for sulfide concentration using the methylene blue method (Strocchi et al., 1992). The other fraction was digested with 2 mL metal-free 20% nitric acid at 65 °C for 24 h. The solution was filtered through a 0.22 μm sterilized and washed membrane filter, and the total iron concentration was measured using inductively coupled plasma mass spectroscopy (ICP-MS, NexION 300X, China) (Zhang et al., 2024). All results were normalized and expressed as sulfide and iron contents per milligram of soluble protein.

### 2.5 XRD, TEM, and NanoSIMS analyses

The XRD was used to analyze the mineralogy of the synthetic mackinawite with a Rigaku Smart lab X-ray powder diffractometer (Rigaku, Japan) employing Cu Kα radiation at 40 kV and 200 mA. The sample was scanned over the 2θ range of 3–75° stepping at 0.04° 2θ with a counting time of 1 s per step (Zhang et al., 2024).

The TEM was used to characterize the original synthetic mackinawite and the mackinawite with either 250 μM Fe^2+^ or HS^−^ in base salt medium. TEM was also used to visualize mineral-cell associations from the culture experiments. Logarithmic-phase MmS2 cells, grown in either 50 μM FeS_Mack_ + 250 μM Fe^2+^ (high-Fe^2+^) or 50 μM FeS_Mack_ + 250 μM HS^−^ (high-HS^−^), were collected and centrifuged at 4,000 g for 10 min. The cell pellet was fixed in 2.5% glutaraldehyde at 4 °C for 4 h, followed by dehydration through a graded ethanol series and final suspension in absolute ethanol. Final samples were dropped onto carbon-coated copper grids and imaged using a JEOL JEM-2100F TEM equipped with EDS and electron diffraction at 200 kV accelerating voltage. A field-emission TEM (JEM-F200-SEI, Japan) equipped with an SEI secondary-electron detector was used for secondary electron imaging. For TEM observations of intracellular particles, 70-nm sections were cut using an ultramicrotome (Leica Microsystems EM UC7, Germany) and collected on copper grids with a carbon support film (China Jinxin Optical Instrument Co.). Detailed procedures regarding fixation, dehydration and embedding procedure were described in the Supplementary Material 1.2. Sections were visualized using a JEM-1400Flash TEM equipped with EDS (JEOL, Japan) at 80 kV accelerating voltage equipped with a 200,000-pixel CMOS camera XAROSA (EMSIS, Germany).

For nanoSIMS analysis, 500-nm thick sections were cut and collected on single-crystal silicon wafers (Newstar BaiRui, China). NanoSIMS 50 L (CAMECA, France) was operated in scanning mode for SIMS imaging. A ^12^C^14^N-map was created first to visualize cell outlines. Fe was detected as ^56^Fe^16^O, S as ^32^S, and Fe-S co-occurrence as FeS. The ion probe scanned the sample through a 256 × 256 pixel raster to generate secondary ion images, with a dwell time of 10 milliseconds per pixel. Images were processed using ImageJ software^1^, and elemental data from regions of interests (ROIs) were collected using ImageJ with the Open MIMS plugin^2^.

### 2.6 RNA and protein extraction, sequencing, and analysis

To investigate cellular responses to FeS_Mack_ under high-Fe^2+^ and high-HS^−^ conditions, transcriptomic and proteomic analyses were performed on triplicate samples. Cells from 50 mL of culture (equivalent to a total of ~4 × 10^10^ cells) were collected under anaerobic conditions in the logarithmic phase for RNA and protein extractions. Detailed procedures for RNA and protein analysis, including sequencing and data processing, can be found in the Supplementary Materials 1.3, 1.4. In short, total RNA from MmS2 cells was extracted using TRIzol (Invitrogen, United States) with DNase I treatment (TaKaRa, Japan), quantified with Qubit RNA HS assay kit and fluorometer (Invitrogen, United States), and quality-checked by NanoDrop (Thermo Fisher Scientific, United States). RNA-seq was performed on the Illumina platform; rRNA was removed by Bowtie2, low-quality reads filtered by fastp (Chen et al., 2018), and transcripts quantified with Salmon (Patro et al., 2017). Differential expression was analyzed using DESeq2 (log2FC > 1/<−1, padj <0.01) (Love et al., 2014).

For proteomics, cells were rinsed with PBS anaerobically, and lysed in urea/thiourea buffer in the presence of protease inhibitors. Protein concentration was measured by using Qubit. Twenty μg of total protein were separated by SDS-PAGE, and digested with trypsin. The resulting peptides were desalted using RP-C18 StageTips. The desalted peptides were dissolved in 10 μL of 0.1% formic acid and 1 μL was injected for analysis (Li et al., 2025; Zhou et al., 2024). Nano liquid chromatography (LC)-Orbitrap mass spectrometry (MS) was used for peptide analysis (Cox and Mann, 2011). Proteins were identified and quantified using MaxQuant version 2.7.5.0 (Tyanova et al., 2016a). Peptide and protein identifications were controlled at a false-discovery rate of <1%, and proteins supported by at least one unique peptide were retained. Potential contaminants, reverse-database matches, and proteins identified only by site were removed. Proteins quantified in at least two of the three biological replicates in at least one treatment group were retained. After log_2_ transformation, missing LFQ intensity values were imputed separately for each sample from a normal distribution using the Perseus (Version 2.2.1) parameters of width = 0.3 and downshift = 1.8 (Tyanova et al., 2016b). The values were median-centered across samples and back-transformed to the original intensity scale. Differential protein abundance was subsequently analyzed using DESeq2 (Love et al., 2014), with log_2_FC > 1/<−1 and a Benjamini-Hochberg-adjusted *p*-value (padj) < 0.01 as the significance criteria.

## 3 Results and discussion

### 3.1 Mackinawite characterization

The XRD pattern revealed a set of characteristic peaks of nanocrystalline mackinawite (Figure 1 and Supplementary Table 1). In the base salt medium, TEM images revealed some dissolution of the original mackinawite along the edge, with loss of lattice fringes (Figure 2A). However, in the presence of either 250 μM Fe^2+^ or HS^−^, the mackinawite did not show any dissolution (Figures 2B,C). EDS analysis revealed a nearly stoichiometric Fe/S ratio for mackinawite (Figure 2). Nonetheless, all three samples exhibited a typical structure and composition of nanocrystalline mackinawite.

**Figure 1:**
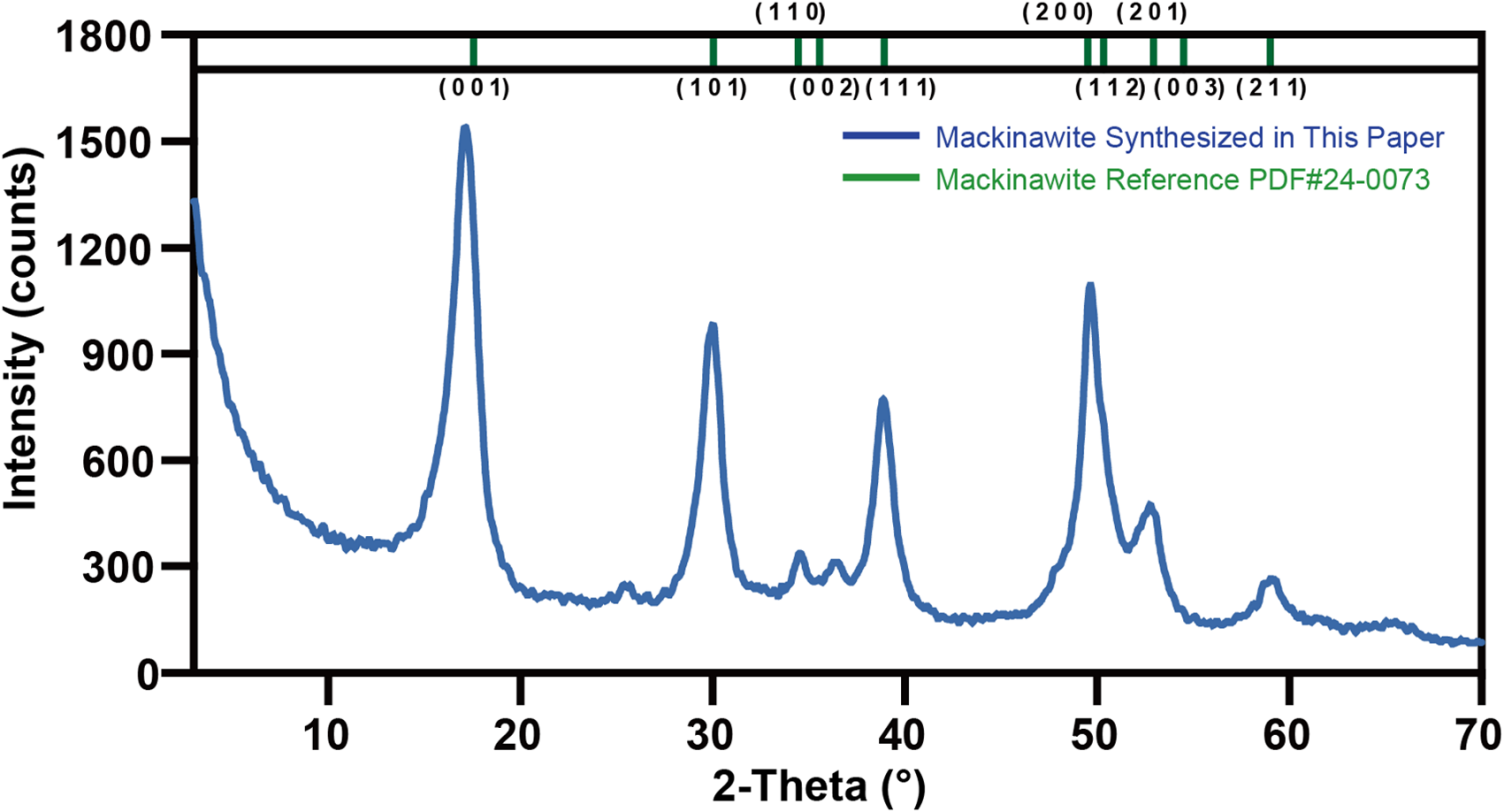
X-ray diffraction patterns of synthesized mackinawite.

**Figure 2:**
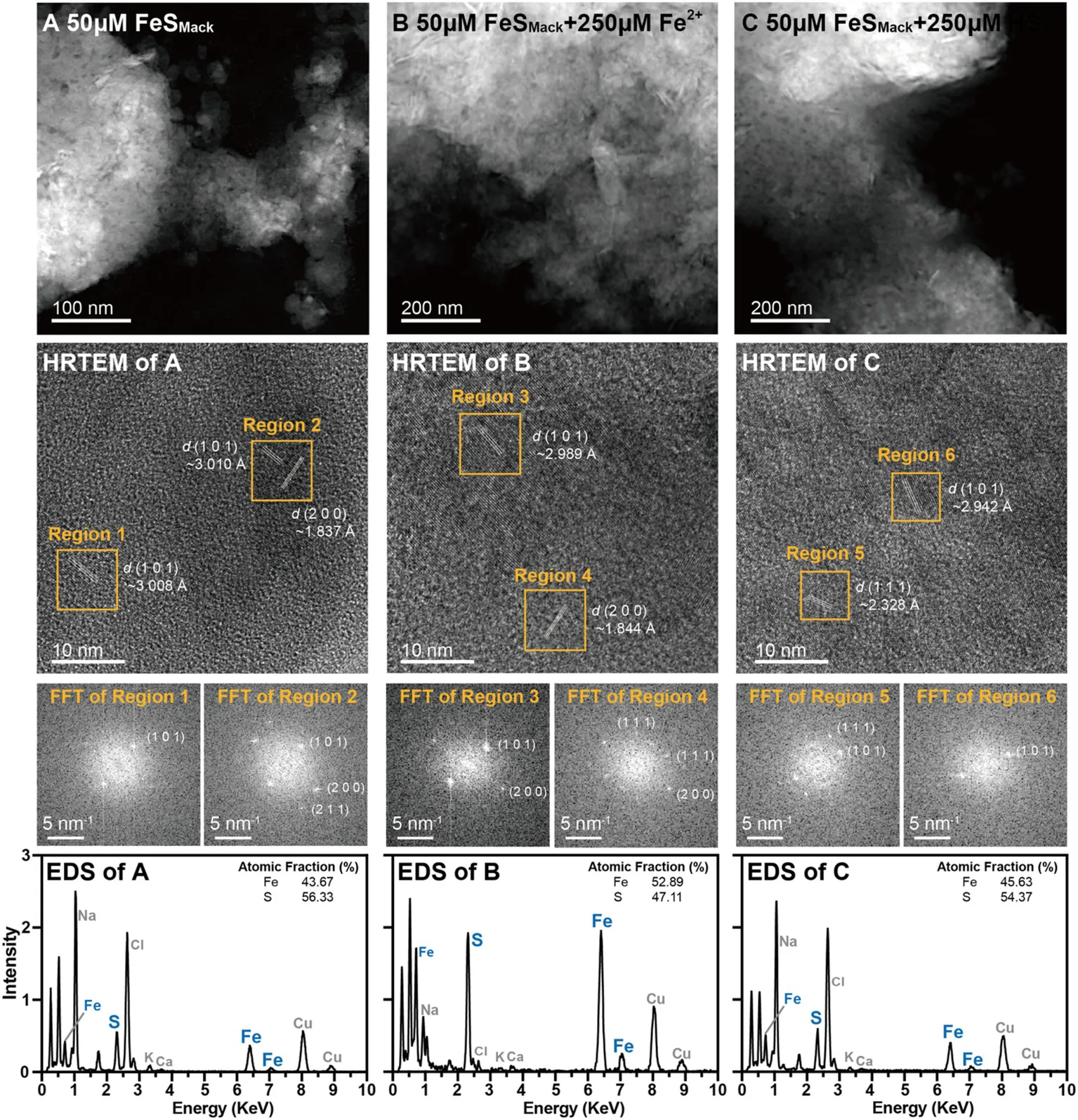
Transmission electron microscopy (TEM), high-resolution TEM (HRTEM), fast Fourier transform (FFT), and energy-dispersive X-ray spectroscopy (EDS) analyses of mackinawite under different Fe^2+^ and HS^−^ conditions. The TEM images in the top row show **(A)** 50 µM FeS_Mack_ alone, **(B)** 50 µM FeS_Mack_ supplemented with 250 µM Fe^2+^, and **(C)** 50 µM FeS_Mack_ supplemented with 250 µM HS^−^. The corresponding HRTEM images, FFT patterns, and EDS spectra are shown below each TEM image in the same column. TEM images show the general morphology, while HRTEM images reveal lattice fringes corresponding to specific crystallographic planes. Together, these analyses support the identification of the material as nanocrystalline mackinawite.

### 3.2 Iron and sulfur speciation in mackinawite-Fe^2+^-HS^−^ systems

In the mackinawite-Fe^2+^-HS^−^ system used for the subsequent culture experiments, there were four possible species of Fe and S, including Fe^2+^, HS^−^, FeS_aq_, and FeS_Mack_, with their relation proportions depending on the specific conditions. When 50 μM FeS_Mack_ was dissolved in the base salt medium, FeS_Mack_ partially dissolved (Figure 3 and Supplementary Table 2), consistent with the TEM observations (Figure 2). However, the dissolution of FeS_Mack_ was inhibited when either Fe^2+^ or HS^−^ was added. When the Fe^2+^ to FeS_Mack_ ratio reached > 3, FeS_Mack_ did not dissolve to release any aqueous HS^−^ (Figure 3 and Supplementary Table 2). Likewise, when the HS^−^ to FeS_Mack_ ratio reached > 3, no detectable aqueous Fe^2+^ was observed from FeS_Mack_ dissolution. These findings suggest that addition of Fe^2+^ and HS^−^ inhibited the dissolution of FeS_Mack_. Geochemical modeling with MINTEQ supported these findings, showing that the saturation index of mackinawite increased when Fe^2+^ or HS^−^ were added (Supplementary Table 3). In addition, other carbonates (such as dolomite, rhodochrosite) and sulfides (NiS, covellite, CoS, chalcopyrite) were oversaturated because of the presence of metals in the base salt medium. However, supersaturation indicates only the thermodynamic potential for precipitation and does not confirm mineral formation. TEM-EDS did not detect the diagnostic elements of these predicted phases, while HRTEM and FFT results were consistent with FeS_Mack_ (Figure 2). Therefore, although their presence in minor amounts cannot be completely excluded, these phases were unlikely to have contributed substantially to the characterized FeS_Mack_.

**Figure 3:**
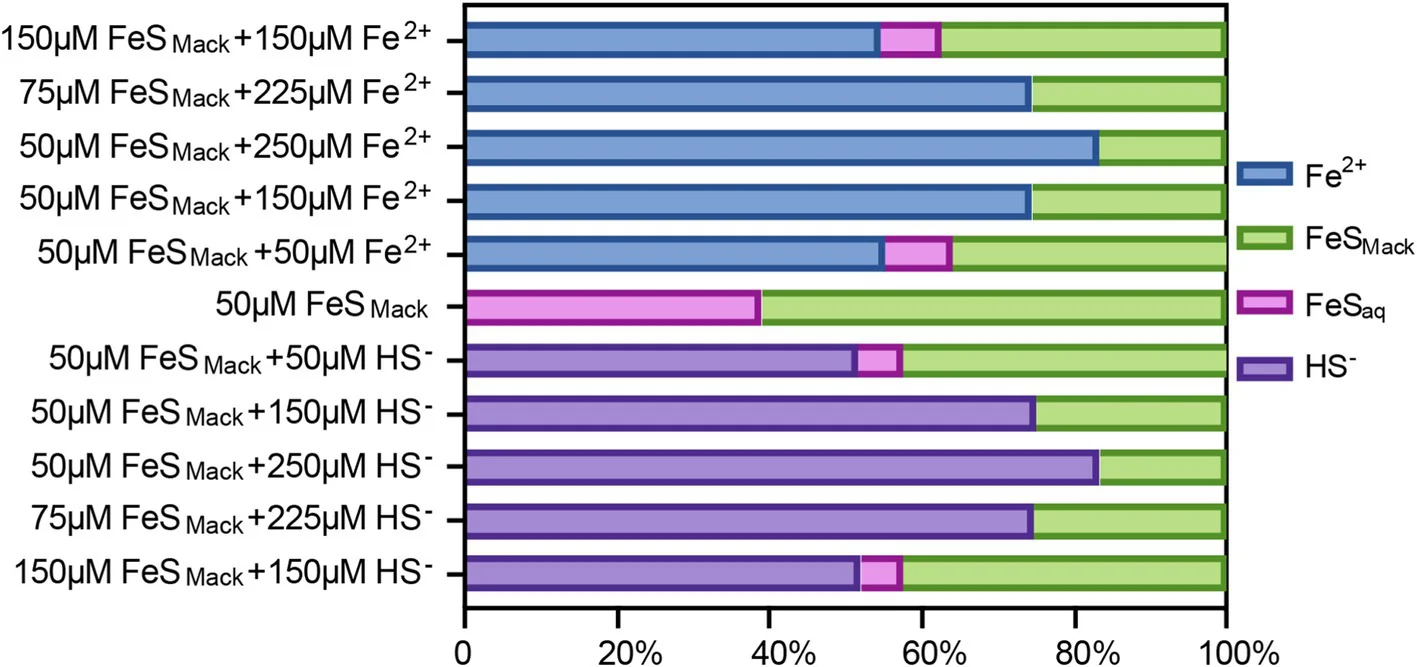
Iron and sulfur speciation in mackinawite-Fe^2+^-HS^−^ systems. In the experiments, Fe^2+^ and HS^−^ were measured in the experimental supernatant and FeS_aq_ was calculated as the difference between the measured Fe^2+^ or HS^−^ concentrations and the actual amount of Fe^2+^ or HS^−^ added (see text for details). The stacked bars show the relative molar proportions of the components; the corresponding absolute concentrations (μM; mean ± SD) are provided in Supplementary Table 2.

FeS_aq_ comprises soluble (FeS)_n_ clusters containing up to approximately 150 FeS units (Rickard, 2006), which can pass through a 0.22 μm filter and are difficult to measure directly. Under our experimental conditions, it should have been included as part of measured Fe^2+^ or HS^−^. Nevertheless, its concentration can be calculated. For instance, when 150 μM Fe^2+^ was added to 150 μM FeS_Mack_, the measured Fe^2+^ concentration was 179.13 ± 2.25 μM (Supplementary Table 2). The difference (29.13 ± 2.25 μM) between the measured Fe^2+^ (179.13 ± 2.25 μM) and added Fe^2+^ (150.00 μM) should be in the form of FeS_aq_ from FeS_Mack_ dissolution. Similarly, the measured HS^−^ concentration of 23.69 ± 4.78 μM should represent FeS_aq_ from the dissolution of FeS_Mack_ (Supplementary Table 2) and was similar to 29.13 ± 2.25 μM, within the errors. For the purpose of presenting FeS_aq_, an average of these two values was obtained (Supplementary Table 2) and plotted (Figure 3). Considering the Fe^2+^ concentrations in natural environment and pure cultures (Holland, 1973; Whitman et al., 1986), we selected 50 μM FeS_Mack_ with varying Fe^2+^ and HS^−^ concentrations for the subsequent culture experiments.

### 3.3 Growth and methanogenesis of *Methanococcus maripaludis* S2 on mackinawite under different Fe^2+^ and HS^−^ conditions

The presence of mackinawite particles induced cell clumping (Supplementary Figures 1A–D), to result in inaccurate cell count and optical density measurement (Supplementary Figure 1E), but did not interfere with DNA measurement (Supplementary Figure 1F). Previous studies have shown that culture conditions can influence cell size and cell density in MmS2, potentially affecting DNA-based biomass estimates (Payne et al., 2023). However, our microscopic analysis revealed no significant difference in cell size between different treatment groups (Supplementary Figures 1C,D). Nonetheless, considering other potential influencing factors, DNA concentration in this study should be interpreted as an indicator of relative biomass changes rather than absolute cell numbers.

MmS2 did not grow and produce CH_4_ with 50 μM Fe^2+^ only (Figures 4A,B). In contrast, MmS2 grew and produced CH_4_ with 50 μM HS^−^ alone (Figures 4C,D), suggesting the greater importance of HS^−^ than Fe^2+^, consistent with a previous study for *Methanococcus voltae* (Payne et al., 2021b). When a range of Fe^2+^ (0–250 μM) was added to 50 μM FeS_Mack_, MmS2 exhibited similar growth and methanogenic activity (Figures 4A,B). Similarly, addition of 0–250 μM HS^−^ to 50 μM FeS_Mack_ resulted in similar growth and methanogenesis, all of which were higher than 50 μM HS^−^ alone (Figures 4C,D). When the concentration of FeS_Mack_ was changed to 37.5, 75 and 150 μM, while maintaining the same total Fe/S ratio, the growth and methanogenesis trends remained the same (Supplementary Figure 2). This finding is consistent with the comparable cell growth and methane production reported for methanogen supplied with either FeS_Mack_ or equivalent dissolved Fe^2+^/HS^−^ (Payne et al., 2021b). Our results further showed that MmS2 maintained growth and methanogenic activity under high-Fe^2+^ and high-HS^−^ conditions that inhibited the dissolution of FeS_Mack_, and highlighted the ability of MmS2 to adapt to fluctuations in Fe^2+^, HS^−^ and FeS_Mack_ levels.

**Figure 4:**
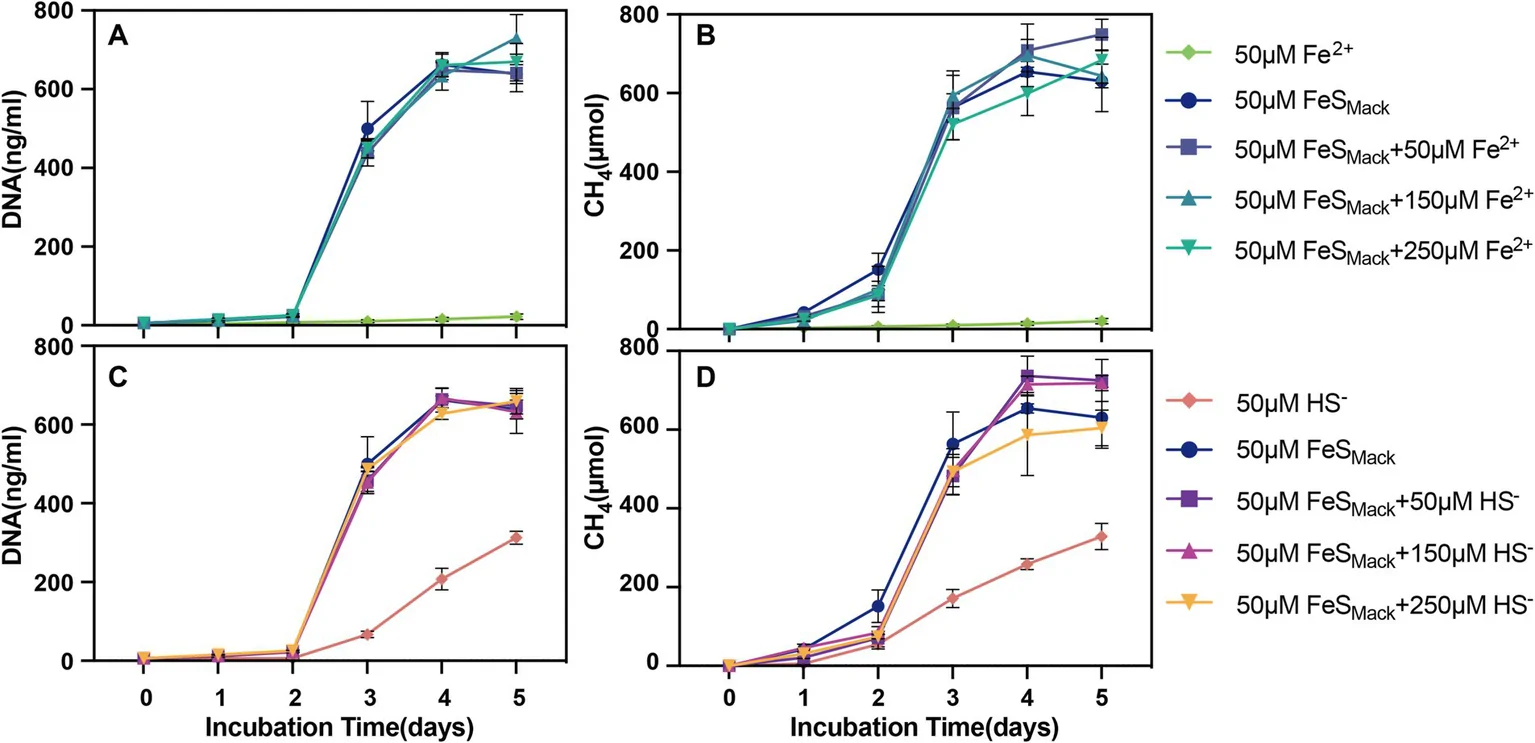
Growth and methanogenic activity of *Methanococcus maripaludis* S2 at different concentrations of Fe^2+^ or HS^−^. The methanogenic activity and growth were monitored by quantifying changes of DNA **(A,C)** and methane production **(B,D)** over time. The data represent averages with standard deviations from 3 experimental replicates.

Coupled Fe-S speciation and cell growth/methanogenesis were examined in detail. In the presence of 50 μM FeS_Mack_, approximately 21 μM HS^−^ and 18 μM Fe^2+^ were measured in the supernatant (Figure 5), likely in the form of FeS_aq_. During the log phase, HS^−^ level gradually decreased to approximately 4 μM (Figures 5B,D), but Fe^2+^ level remained stable or even slightly increased (Figures 5A–C). Indeed, the much greater amount of decrease of aqueous HS^−^, relative to Fe^2+^, corresponded to a much higher amount of intracellular HS^−^ relative to Fe^2+^ (Figure 6). After adding 50 μM Fe^2+^ to 50 μM FeS_Mack_, approximately 9 μM HS^−^ was measured, which should be in the form of FeS_aq_ due to excess Fe^2+^, but this level of HS^−^ was quickly consumed (Figure 5B), while Fe^2+^ level remained stable (Figure 5A). Conversely, after adding 50 μM HS^−^ to 50 μM FeS_Mack_, ~9 μM Fe^2+^ was present as FeS_aq_, and its level slowly decreased over time (Figure 5C) but HS^−^ was nearly consumed over the same period. These results suggested that when low levels of either aqueous Fe^2+^ or HS^−^ were added, MmS2 was able to mobilize either S or Fe from FeS_aq_ to sustain its growth and methanogenesis, while maintaining intracellular sulfide and Fe^2+^ concentrations at levels comparable to the 50 μM FeS_Mack_ (Figure 6).

**Figure 5:**
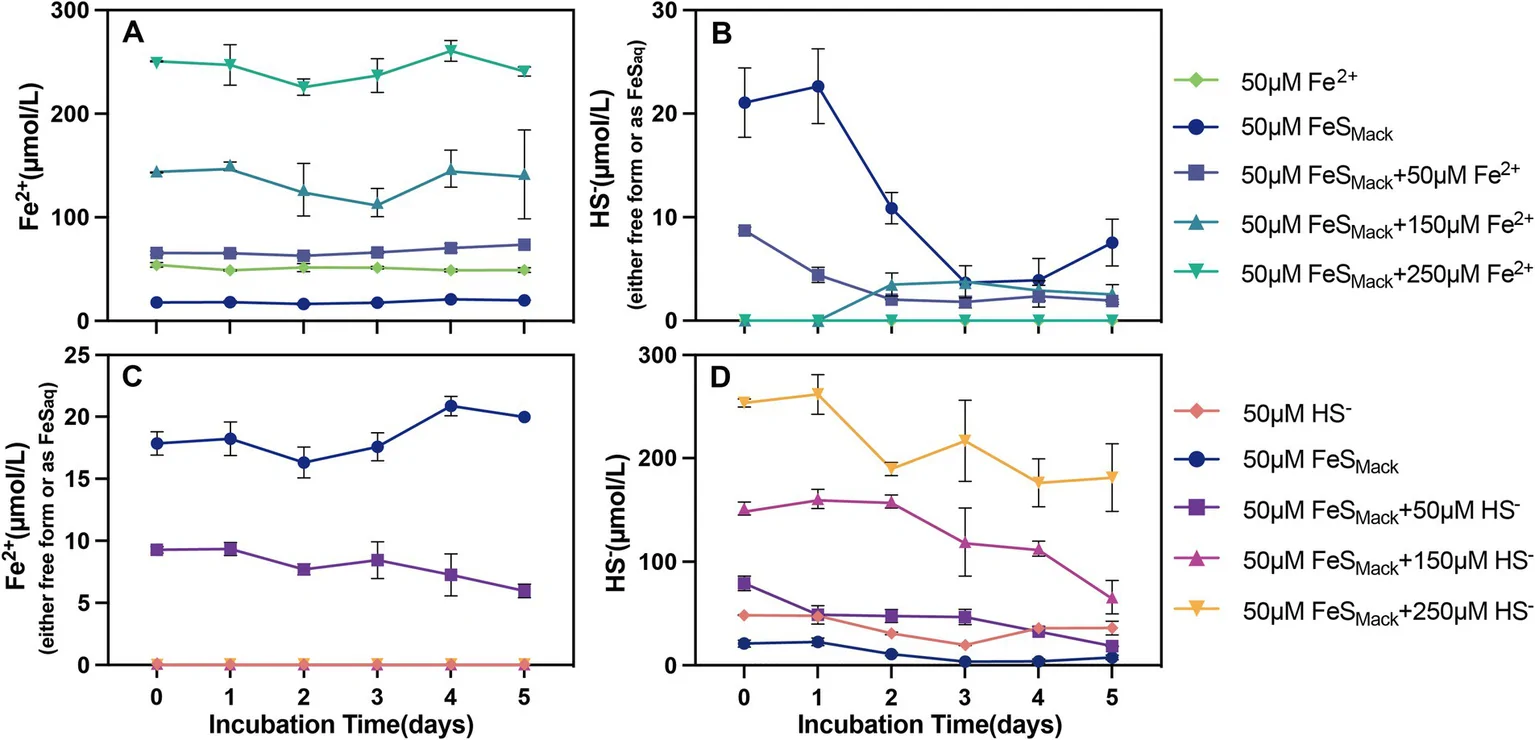
Concentrations of Fe^2+^ and HS^−^ in the culture medium during the growth of *Methanococcus maripaludis* S2. Time-course changes of Fe^2+^ **(A)** and HS^−^ **(B)** concentrations under rich-Fe^2+^ conditions. Time-course changes of Fe^2+^ **(C)** and HS^−^ **(D)** concentration under rich-HS^−^ conditions. At high-Fe^2+^ or high-HS^−^ concentrations, low concentrations of HS^−^ or Fe^2+^ may exist in either free form or FeS_aq_. In the 150 μM and 250 μM Fe^2+^ treatments, HS^−^ was below the detection limit and denoted as 0 **(B)**. Likewise, Fe^2+^ concentration in the treatments of 50 μM HS^−^, 50 μM FeS_Mack_ + 150 μM HS^−^ and 50 μM FeS_Mack_ + 250 μM HS^−^ were below the detection limit and denoted as 0 **(C)**. The data represent averages with standard deviations from 3 experimental replicates. The abiotic controls did not show substantial changes in either Fe^2+^ or HS^−^ concentrations and are provided in Supplementary Table 4.

**Figure 6:**
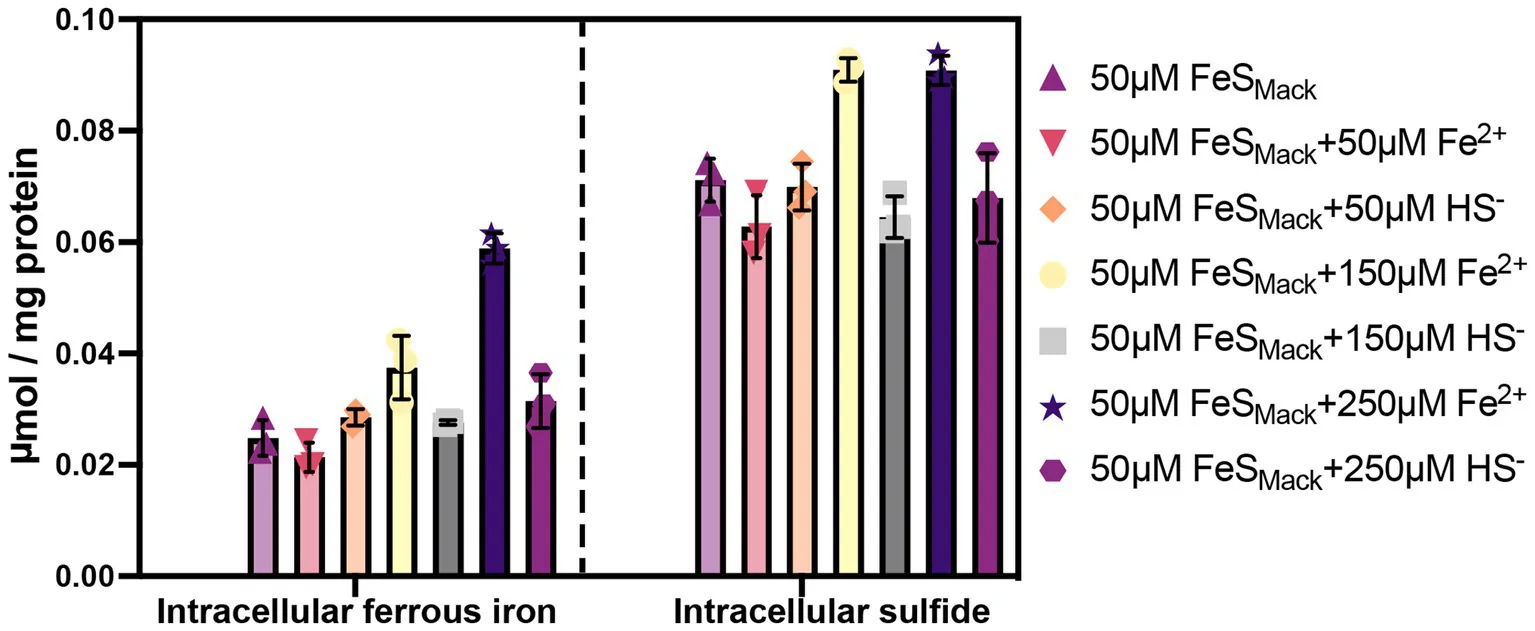
Intracellular ferrous iron and sulfide contents under high-Fe^2+^ and high-HS^−^ conditions. The data represent averages with standard deviations from 3 experimental replicates. The ferrous iron and sulfide levels in the initially inoculated cells were below the detection limit.

When the Fe^2+^ to FeS_Mack_ ratio reached 3:1, FeS_aq_ was either non-present or at a low level (Figure 5B and Supplementary Table 2), yet there was significant cell growth and methanogenesis (Figures 4A,B), suggesting that FeS_Mack_ likely provided a source of S. Conversely, when the HS^−^ to FeS_Mack_ ratio was 3:1, there was no FeS_aq_ (Figure 5C and Supplementary Table 2), yet there was still significant growth and methanogenesis (Figures 4C,D), in this case, FeS_Mack_ likely provided a source of Fe. When either the Fe^2+^ to FeS_Mack_ ratio or the HS^−^ to FeS_Mack_ ratio was further increased to 5:1, there was no FeS_aq_ throughout the entire growth period (Figures 5B,C and Supplementary Table 2). In these cases, either Fe or S required for growth and methanogenesis should have come from the FeS_Mack_. When three- or five-fold excess HS^−^ was added to 50 μM FeS_Mack_, the intracellular enrichment of Fe^2+^ and HS^−^ remained similar to those with the 50 μM FeS_Mack_ alone, again suggesting that the demand for Fe^2+^ was low (Figure 6). In contrast, addition of three- or five-fold excess Fe^2+^ led to greater intracellular accumulations of both Fe^2+^ and HS^−^. These results again suggested that under conditions of replete Fe^2+^ but low HS^−^, the cells were able to take up HS^−^ from FeS_Mack_ for essential metabolic processes such as Fe-S cluster biogenesis.

### 3.4 Visual evidence for intracellular accumulation of FeS nanoparticles under high-Fe^2+^ condition

The above result showed that MmS2 acquired Fe and S from FeS_Mack_ to meet its metabolic needs. To further verify and visualize this intracellular enrichment, two experimental groups, 50 μM FeS_Mack_ with either 250 μM Fe^2+^ (high-Fe^2+^) or 250 μM HS^−^ (high-HS^−^), were selected for high-resolution imaging (Figure 7 and Supplementary Figure 3). TEM results revealed that MmS2 cells grown under the high-Fe^2+^ condition contained many black intracellular particles, approximately 10–30 nm in diameter (Figure 7A), evenly distributed both within the cytoplasm and along the cell periphery. However, when grown under the high-HS^−^ condition, cells exhibited fewer and smaller intracellular granules, with some larger granules near the surface (Figure 7B). TEM-EDS analysis identified that these particles were made of Fe and S (Figures 7C,D), with a similar composition to that of extracellular particles (Figure 7E). Secondary electron and TEM images, along with compositional analysis, provided additional evidence for the presence of intracellular particles under high-Fe^2+^ conditions (Supplementary Figure 4).

**Figure 7:**
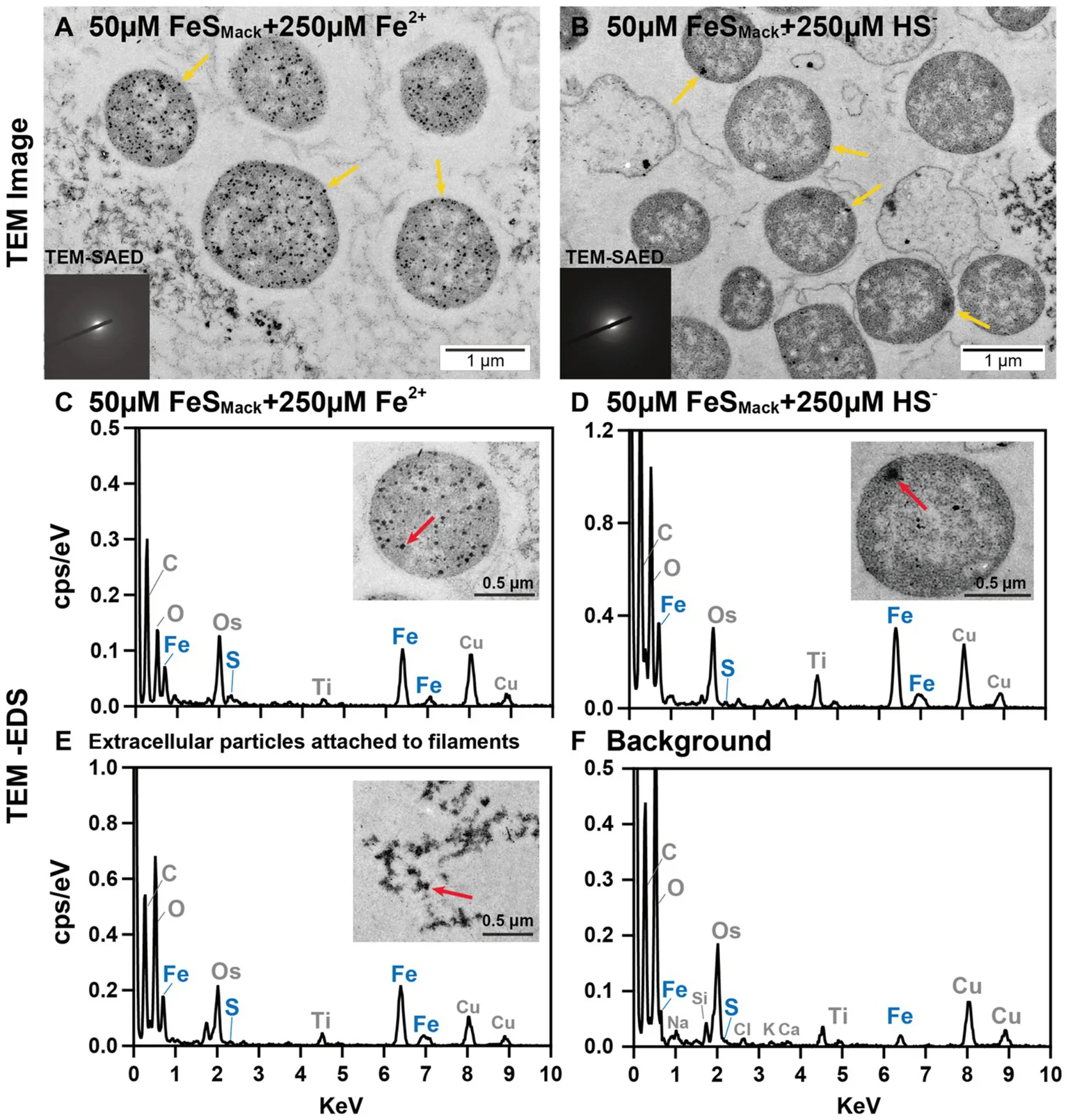
TEM images of *Methanococcus maripaludis* S2 cell sections and TEM-EDS elemental analysis of intracellular particles. **(A,B)** Seventy-nm thin sections of MmS2 cells collected in 50 μM FeS_Mack_ with either 250 μM Fe^2+^ **(A)** or 250 μM HS^−^ **(B)** revealed the presence of intracellular nanoparticles. Particles located at the cell periphery are indicated by yellow arrows. **(C–E)** TEM-EDS analysis was performed on the intracellular particles in both experimental groups as well as on the minerals attached to the filaments to determine their elemental composition. **(F)** EDS spectrum of background (resin) showing Cu from the grid, Ti from the specimen holder, C and O from agar, resin, and mixed biological components, and Os from osmium tetroxide used for fixation and staining.

NanoSIMS imaging revealed distinct differences in the intracellular distribution of Fe-S in different treatments (Figure 8). Under high-Fe^2+^ conditions, ^56^Fe^16^O S and ^32^S signals were markedly enhanced (Figures 8A,D,G). In contrast, under high-HS^−^ conditions, intracellular ^56^Fe^16^O S and ^32^S signals were considerably weaker, with some enrichment detected at the cell periphery (Figures 8B,E,D). Element ratio analysis revealed that the ^56^Fe^32^S/^12^C^14^N ratios were significantly higher in the high-Fe^2+^ conditions than in the high-HS^−^ conditions (Figures 8C,F,I), consistent with the intracellular Fe and S enrichment under high-Fe^2+^ conditions (Figure 6). These findings suggested that under the high-Fe^2+^ condition, the cells may have sensed sulfur limitation and acquired it from FeS_Mack_. Throughout this study, these intracellular particles are therefore referred to as Fe- and S-rich particles based on their elemental composition. No interpretable electron diffraction pattern was obtained, suggesting that the particles may be amorphous and their specific mineral phase could not be determined (Supplementary Figure 4C).

**Figure 8:**
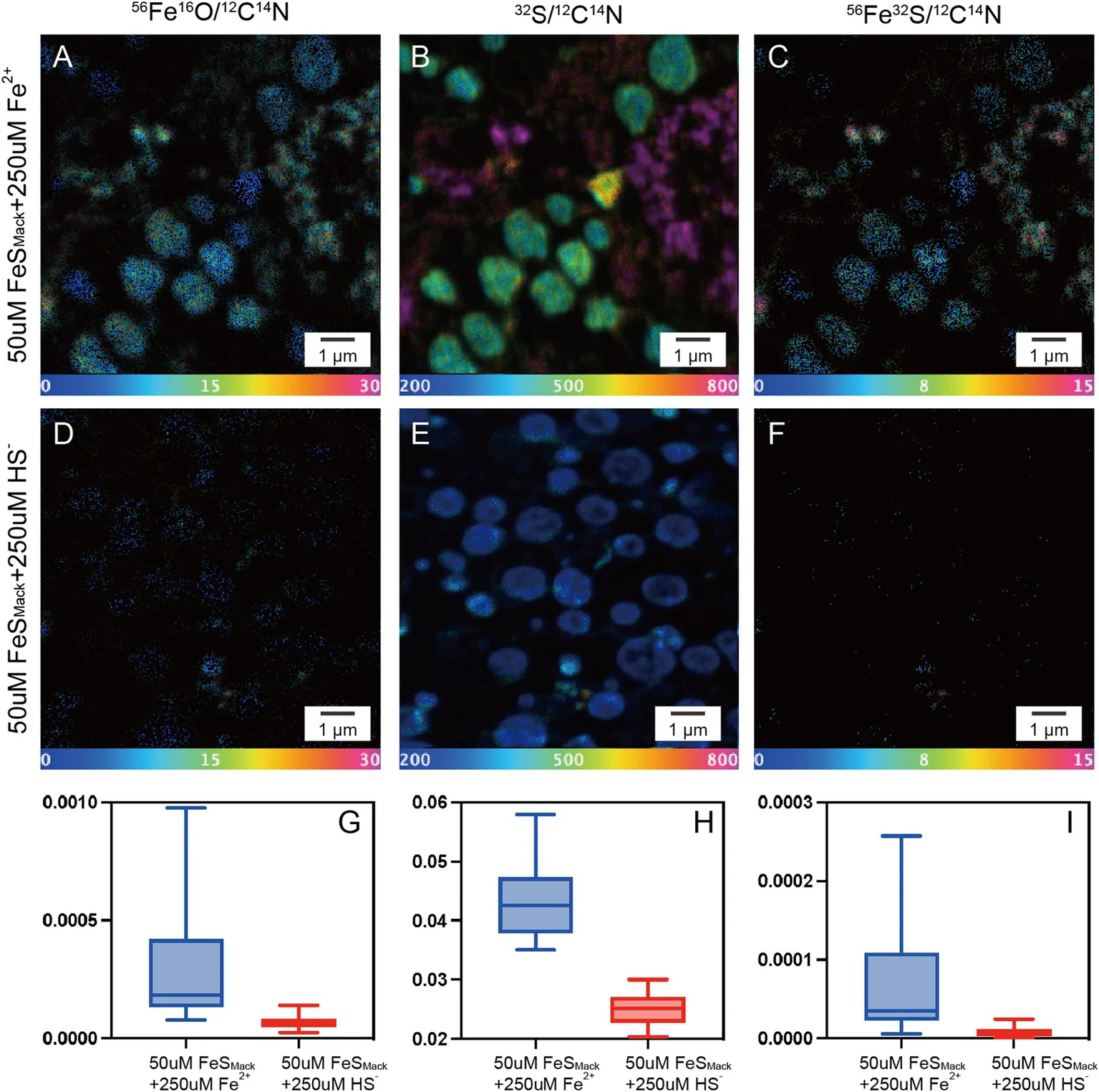
NanoSIM images and relative intracellular contents of iron, sulfur, and iron–sulfur compounds. 500 nm sections of MmS2 cells were collected in 50 μM FeS_Mack_ with either 250 μM Fe^2+^ **(A–C)** or 250 μM HS^−^ **(D–F)**. ^56^Fe^16^O/^12^C^14^N denotes intracellular Fe **(A,D)**, ^32^S/^12^C^14^N denotes intracellular S **(B,E)**, and ^56^Fe^32^S/^12^C^14^N denotes the locations where Fe and S co-occur **(C,F)**. The relative ^56^Fe^16^O/^12^C^14^N, ^32^S/^12^C^14^N and ^56^Fe^32^S/^12^C^14^N content in each cell within the scanned area **(G–I)**. The color bands represent the intensity of the scan response. Data in all figures are from the same embedded sample, but different slices were used due to different thickness requirements for TEM and NanoSIMS.

Under the high-Fe^2+^ condition, FeS_Mack_ was the only available source of sulfur. The intracellular accumulation of Fe- and S-rich particles may reflect a response of MmS2 to sulfur limitation, although whether these particles represent a mobilizable reserve or a sequestration product remains unclear. In contrast, under the high-HS^−^ condition when cells could effectively utilize aqueous HS^−^, they only needed a small amount of Fe from FeS_Mack_ to meet its Fe demand, which was stored as Fe- and S-rich particles at the edge of the inner membrane (Figure 7B). These Fe- and S-rich particles differ from magnetosomes in that they lack an ordered arrangement (Pérez-Huerta et al., 2022), and they are also distinct from intracellular Fe- and S-rich particles formed through the reduction of S^0^ (Clarkson et al., 2010; Vaccaro et al., 2017) or SO_4_^2−^ (Deng et al., 2020; Jones et al., 1976). Under anaerobic conditions, the utilization of FeS_Mack_ by methanogens is unlikely to involve redox processes, as Fe(II)-S(II) is already in its reduced state, and there is no mechanism for further biological oxidation or reduction (Kracke et al., 2015). Therefore, it remained unclear how intracellular Fe- and S-rich particles formed under the high-Fe^2+^ condition, but with two possible scenarios: (1) through direct assimilation of extracellular FeS_Mack_ particles to meet S and Fe requirements; (2) import of solubilized Fe and S from FeS_Mack_ and reprecipitation of Fe- and S-rich particles inside the cell for storage. Detailed physiological characterization, identification of transport mechanisms, in combination with loss-of-function study, are required to delineate the specific formation mechanisms of intracellular Fe- and S-rich particles.

### 3.5 Coordinated metabolic remodeling and energy reallocation of MmS2 under the high-Fe^2+^ condition

To investigate how MmS2 maintained comparable growth and methane production despite the intracellular accumulation of Fe- and S-rich particles, the transcriptomic and proteomic profiles of the logarithmic phase cells were compared under the high-Fe^2+^ and high-HS^−^ conditions. PCA separated the high-Fe^2+^ and high-HS^−^ transcriptomes along PC1 (77.3%), with high correlations among the High-Fe^2+^ replicates (*r* = 0.982–0.987). Despite greater replicate variability, the proteomic profiles retained an overall treatment-associated separation (Supplementary Figure 5). Among the 1,722 genes analyzed, 618 differentially expressed genes were identified, with 42.20% of them being upregulated and 57.80% downregulated. In the analysis of 762 detected proteins, 107 were differentially expressed proteins, with 39.25% upregulated and 60.75% downregulated. Proteomic profiles reflected the cellular capabilities that were realized at the time of sampling, whereas transcriptomic reflected potential regulatory states or future cellular demands. Differentially expressed genes and proteins under high-Fe^2+^ relative to high-HS^−^ conditions were indicative of functional reprogramming and adaptive responses associated with intracellular FeS enrichment (Figure 9 and Supplementary Table 5).

**Figure 9:**
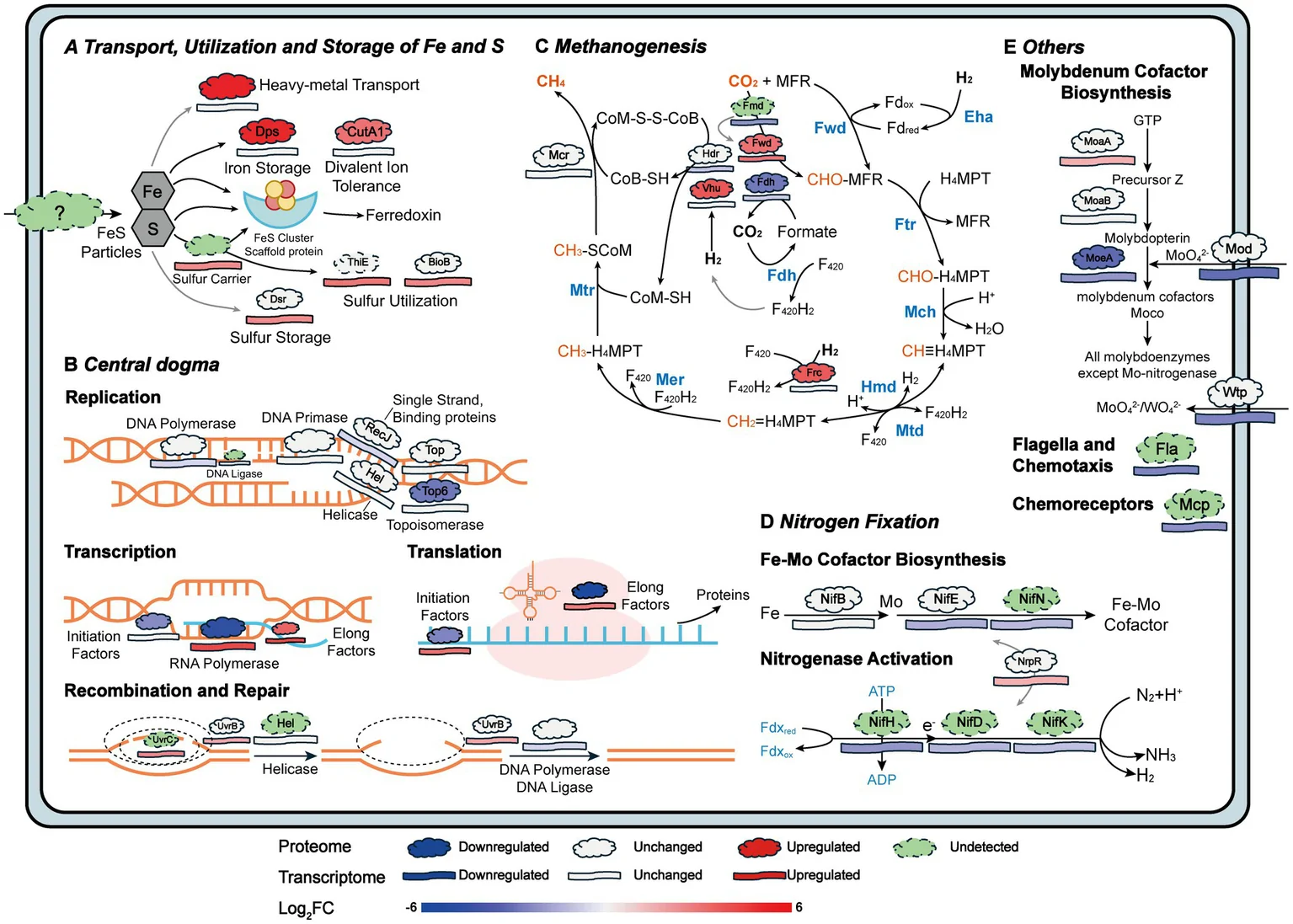
The proteome and transcriptome of *Methanococcus maripaludis* S2 under high-Fe^2+^ conditions relative to high-HS^−^ conditions. Curved shapes in dark blue, gray, red and green colors denote downregulated, unchanged, upregulated, and undetected proteins in the proteome, respectively. Horizontal bar shapes in dark blue, gray, and red denote downregulated, unchanged, upregulated genes in the transcriptome, respectively. **(A)** Representative proteins in *Methanococcus maripaludis* S2 are categorized by their functional roles. Proteins involved in iron and sulfur transport, utilization, and storage include Dps (iron storage), CutA1 (divalent ion tolerance), ThiE and BioB (sulfur utilization), Dsr (sulfur storage), as well as FeS cluster scaffold proteins and sulfur carriers for Fe-S cluster assembly and sulfur transport. **(B)** Central dogma-related proteins encompass DNA polymerase, DNA primase, RecJ (single-strand binding and repair), helicase (Hel), topoisomerases (Top/Top6), DNA ligase, RNA polymerase, transcription/translation initiation and elongation factors, and recombinational repair proteins UvrB/UvrC. **(C)** Methanogenesis-associated proteins include Mcr (methyl-coenzyme M reductase), Hdr (heterodisulfide reductase), Vhu (hydrogenase), Fdh (formate dehydrogenase), Fmd/Fwd (formylmethanofuran dehydrogenases), Frc (F420-dependent reductase). **(D)** Nitrogen fixation proteins include NifB/NifE/NifN (Fe-Mo cofactor biosynthesis), NifH/NifD/NifK (nitrogenase activation), the regulatory protein NrpR. **(E)** Other functional proteins comprise MoaA/B/C/E, Mod (molybdenum cofactor biosynthesis), and Wtp (tungstate and molybdate transporter), Fla. (flagella-related proteins), and Mcp (chemotaxis receptors). Gene or protein IDs and their differential analysis results are provided in Supplementary Table 5.

Proteins associated with sulfur transport and utilization showed minimal differences between the high-Fe^2+^ and high-HS^−^ conditions at the proteomic level (Figure 9A). In contrast, the transcriptome revealed clear upregulation of genes involved in sulfur transport, as well as sulfur utilization pathways such as thiamine phosphate and biotin biosynthesis, together with the putative S^0^-storage gene Dsr (Grimm et al., 2010) in high-Fe^2+^ group. The transcriptomic results revealed a sulfur-related stress response associated with intracellular Fe- and S-rich particles accumulation, which may facilitate sulfur mobilization from these particles. If this interpretation is correct, increased sulfur turnover from Fe- and S-rich particles could release additional free Fe, thereby increasing the intracellular Fe burden. Consistent with this hypothesis, the upregulation of Dps may reflect an enhanced cellular capacity for free Fe storage and detoxification (Chiancone et al., 2004). In addition, a heavy-metal transport ATPase was upregulated at the protein level, which exports Cu^2+^, Zn^2+^, and Co^2+^ (Argüello et al., 2007). Although Fe^2+^ export by this ATPase has not been reported, the combined transcriptomic and proteomic responses suggest that metal-ion homeostasis was perturbed. Similarly, the upregulation of the CutA1 divalent-ion tolerance protein was consistent with a cellular response to metal-ion imbalance, reflecting enhanced tolerance to metal stress (Fong et al., 1995). Together, these observations indicated that the sulfur-dependent methanogen MmS2, when relying on FeS_Mack_ as the sole sulfide source under high-Fe^2+^ conditions, was not merely experiencing passive stress from intracellular FeS accumulation, but was mounting a coordinated response that may support sulfur recycling and adjust its metal-homeostasis network.

The altered metal homeostasis associated with intracellular Fe- and S-rich particles accumulation was accompanied by broad physiological responses in MmS2 (Figure 9B). All DNA replication-related proteins, except for topoisomerase, remained unchanged, indicating limited changes in these proteins at the proteomic level under both high-Fe^2+^ and high-HS^−^ conditions. The transcriptome showed downregulation of two key enzymes, polymerase and DNA-specific exonuclease, which may reflect reduced investment in DNA replication and repair under conditions associated with Fe- and S-rich particles accumulation. There was a noticeable difference between the transcriptomic and proteomic data for DNA transcription and translation related processes, with certain RNA polymerase subunits and translation initiation/elongation factors being upregulated at the transcript level but downregulated at the protein level. This suggested that MmS2 may be reassembling its transcription-translation system to prioritize stress adaptation over standard protein synthesis. Over 30 ribosomal proteins were upregulated at the transcriptional level only, suggesting enhanced transcriptional investment in ribosome biogenesis or remodeling. This transcriptional response may support the preferential translation of stress-response proteins or proteins involved in energy metabolism (Liu and Qian, 2014). In addition, UvrB and UvrC, which are involved in DNA damage verification and dual-incision reactions, were induced at the transcriptional level, suggesting activation of a DNA damage-response pathway (Farnell, 2011). Taken together, the expression patterns observed under conditions associated with Fe- and S-rich particles accumulation were consistent with adjustments in DNA replication, transcription-translation processes, and DNA repair. These responses may contribute to the maintenance of cellular homeostasis, although the underlying mechanisms require further experimental verification.

The intracellular accumulation of Fe- and S-rich particles would disrupt metal homeostasis, prompting MmS2 to reprogram its genetic and metabolic pathways to adapt to these altered conditions (Figure 9C). The molybdenum-dependent formate dehydrogenase (Fdh) is responsible for oxidizing formate to CO_2_ and transferring electrons to the methane production pathway (Boyington et al., 1997; Jormakka et al., 2002). In cells with accumulated FeS, Fdh was downregulated at both the transcriptomic and proteomic levels. This downregulation was likely due to decreased molybdenum availability (Payne et al., 2023). Together with the upregulation of Fwd and Vhu, this expression pattern may also indicate a shift away from formate-dependent methanogenesis (Yoon et al., 2013). Meanwhile, formylmethanofuran dehydrogenase catalyzes the reduction of CO_2_ to formyl-MFR. The molybdenum-dependent Fmd was undetected at the proteomic level, but downregulated at the transcriptomic level, while the tungsten-dependent homolog, Fwd, was significantly upregulated at both omics levels, clearly demonstrating a shift from molybdenum to tungsten dependency. Previous studies have shown that sulfide-rich conditions can decrease Mo bioavailability through the formation of thiomolybdate species, which may in turn limit the activity or expression of Mo-dependent enzymes (Payne et al., 2023). Similarly, under iron-rich conditions, Mo availability may also become limited, leading methanogens to rely on tungsten-dependent enzymes to sustain carbon assimilation and methanogenesis. This shift likely represented an adaptive response to the changing availability of metals under stress. Coinciding with the upregulation of Fwd was the elevation of hydrogenase Vhu. Vhu, Fwd, and the heterodisulfide reductase Hdr can form a physical complex (Costa et al., 2010), forming an electron bifurcation module (Thauer et al., 2008), suggesting that the cell may enhance its H_2_-based electron input module. Despite the ample availability of formate, MmS2 may prioritize the hydrogen-tungsten pathway over formate metabolism under FeS accumulation conditions. This paradox may arise from the reprogramming of the formate hydrogenlyase pathway, which could enable the conversion of formate to H_2_, which then fed electrons into the electron bifurcation system coupled with Fwd (Costa et al., 2010; Kaster et al., 2011). The terminal enzyme in the methane production pathway, methyl-coenzyme M reductase (Mcr), remained stable at both the transcriptomic and proteomic levels. This suggested that, despite the metal homeostasis crisis induced by FeS accumulation, the cell maintained the core machinery of methane production, optimizing energy capture efficiency by prioritizing the integrity of this crucial process.

Under intracellular FeS accumulation, cells exhibited a coordinated reallocation of metabolic and material resources. Nitrogen acquisition and assimilation systems, including the nitrogenase complex, were broadly transcriptionally downregulated, a pattern consistent with the upregulation of the global nitrogen regulator NrpR under nitrogen-replete conditions (Wisedchaisri et al., 2010) (Figure 9D). In parallel, genes involved in energetically demanding motility and chemotaxis, including flagellar (Halte et al., 2025) and methyl-accepting chemotaxis proteins (Schweinitzer and Josenhans, 2010), were downregulated (Figure 9E), suggesting a shift from an actively motile state toward a maintenance-oriented physiology. Several cofactor biosynthetic pathways, including those for the molybdenum cofactor (Figure 9E), the vitamin B12 precursor cobyrinic acid a,c-diamide, and heme precursors, were likewise transcriptionally reduced, consistent with a global attenuation of complex anabolic investment. This transcriptional repression contrasted with selective protein-level reinforcement of core bioenergetic and structural modules. Key electron carriers linked to methanogenesis, including proteins involved in F420 biosynthesis and UbiX-dependent methanopterin side-chain formation, increased in abundance, together with FtsZ, SecG, and glycosyltransferases required for cell division, protein translocation, and envelope biogenesis. This transcript-protein decoupling pattern enables cells to conserve resources while preserving essential energy-conserving and structural capacities under metal-stressed conditions.

### 3.6 Intracellular accumulation of Fe- and S-rich particles under ferruginous conditions: implication for mineral-microbe co-evolution

The ferruginous oceans of the early Archean, widely hypothesized as a cradle of life (Poulton and Canfield, 2011), imposed intense metal and sulfur constraints on primitive metabolisms. Microorganisms dependent on these elements were therefore forced to develop novel survival and diversification strategies. In resource-limited environments, the ability to acquire sulfur from sulfide minerals may have provided an important survival advantage for sulfur-dependent methanogens. Because HS^−^ exhibits a strong affinity for divalent metals, sulfur acquisition inevitably results in the co-uptake of metals and the intracellular formation of metal sulfides. Our data provide evidence that Fe-rich early Earth settings fostered intimate interactions between FeS minerals and microbial cells (Dong et al., 2022). In contrast to modern cultivation systems for methanogens, which are typically characterized by high-HS^−^ but low-Fe^2+^, methanogens cultured under high-Fe^2+^ conditions accumulated substantially greater numbers of intracellular Fe- and S-rich particles than those grown under high-HS^−^ conditions (Figure 7). This phenomenon is consistent with comparative evolutionary observations showing that ancient anaerobic microorganisms, including methanogens and sulfate-reducing bacteria, contained higher intracellular inventories of Fe and S than modern aerobic organisms (Andreini et al., 2017). These findings suggest that early methanogens may have tolerated intracellular mineral formation while adjusting their sulfur-utilization and metal-homeostasis pathways to maintain growth and metabolism.

Despite the pronounced intracellular accumulation of Fe- and S-rich particles, no adverse effects were observed on growth performance, methane production, cellular morphology, or membrane integrity in MmS2. Although direct catalytic roles of intracellular Fe- and S-rich particles cannot yet be experimentally confirmed, integrated proteomic and transcriptomic analyses revealed coordinated changes in the abundance and expression of enzymes involved in core biological processes, indicating metabolic reorganization under the high-Fe^2+^ condition. The accumulation of Fe- and S-rich particles appeared to have driven a strategic reorganization of intracellular resources and energy allocation, in which organic substrates were partially invested in reinforcing cellular architecture. These particles may have reorganized intracellular resource distribution for replication, transcription, translation, and DNA repair, thereby maintaining the robustness of core cellular processes. Crucially, this mineral-cellular process coupling was associated with the maintenance of growth and methanogenesis under altered metal-homeostasis conditions, while the omics profiles suggested the preservation of core energy-conservation pathways. This capacity likely constituted a key survival mechanism in ferruginous early oceans, where fluctuating metal availability and redox instability would otherwise impose severe constraints on energy metabolism.

Over geological timescales, the acquisition and intracellular accumulation of Fe and S may have represented a physiological adaptation strategy for methanogens inhabiting chemically variable environments. Given the central roles of Fe and S in methanogenesis-related enzymes and cofactors, the availability and intracellular regulation of these elements may not only have influenced methanogen adaptation to ferruginous environments but also have affected the coupled cycling of Fe, S, and C at ocean-sediment interfaces. Although the specific metabolic responses of MmS2, a strain isolated from a modern environment, cannot be directly extrapolated to the earliest methanogenic pathways, MmS2 provides an experimentally tractable model for investigating how Fe and S availability constrains methanogen physiology under ferruginous conditions, thereby generating testable hypotheses concerning mineral-microbe interactions in early Earth environments.

## 4 Conclusion

To better understand how Fe- and S-dependent methanogens responded to contrasting ferruginous and locally sulfidic marine conditions that occurred spatially and temporally across the Archean-Proterozoic transition, particularly their mechanism of utilizing FeS_Mack_ in the high-Fe^2+^, low-HS^−^ Archean environment, we used the methanogen *Methanococcus maripaludis* S2 (MmS2) cultured in a growth medium containing FeS_Mack_ under either high-Fe^2+^ or high-HS^−^ conditions. Our results demonstrate that under high-Fe^2+^ conditions, FeS_Mack_ can be effectively utilized by MmS2 to support its growth and methanogenesis. Notably, intracellular Fe- and S-rich particles enrichment under high-Fe^2+^ conditions was accompanied by coordinated changes in the expression of genes and proteins associated with growth regulation, metabolic organization, and energy conservation, potentially reflecting adjustments in intracellular resource allocation. This study provides new insights into mineral-microbe interactions and identifies a response that may represent a strategy by which early-Earth microorganisms coped with fluctuations in metal and sulfur availability, with potential implications for understanding the co-evolution of life and Earth-surface environments.

## Data Availability

The datasets presented in this study can be found in online repositories. The names of the repository/repositories and accession number(s) can be found in the article/Supplementary material.

## Footnotes

1
http://rsb.info.nih.gov/ij/download.html

2
http://www.nrims.hms.harvard.edu/software.php
