# Supplementary material for: Metabolic adaptation of a methanogen to a ferruginous environment associated with intracellular Fe- and S-rich nanoparticles
Source: Front Microbiol. 2026 Oct 7;17:1967823. doi: 10.3389/fmicb.2026.1967823 (PMC13684076; doi:10.3389/fmicb.2026.1967823)
Supplement: Supplementary file 1 [file Supplementary_file_1.docx]

Supplementary Material

# 1 Supplementary Material

## 1.1 Basic salt medium and stock solutions for the medium.

The basic salt medium contains (in one liter): 0.14 g CaCl_2_·2H_2_O, 0.5 g NH_4_Cl, 3.45 g MgSO_4_·7H_2_O, 2.75 g MgCl_2_·6H_2_O, 0.335 g KCl. All these reagents were dissolved in 1 L MQ-H_2_O and purged with pure-N_2_ in a 98°C water bath for 2 hours. The resulting O_2_-free medium was transferred to an anaerobic chamber and allowed to cool, followed by addition of 27 g HCOONa, 1.36 g CH_3_COONa·3H_2_O, and 1 mL 6 N HCl. The solution above was divided into culture bottles according to the volume required for the experiment and sterilized. After cooling, an anaerobic and sterile 0.014 g/L K_2_HPO_4_ and 5 g/L NaHCO_3_ in final concentration was added.

The 100× vitamin stock solution for MmS2 contains (in one liter): 0.01 g pyridoxine hydrochloride, 0.005 g thiamine hydrochloride, 0.005 g riboflavin, 0.005 g niacin, 0.005 g calcium pantothenate, 0.002 g biotin, 0.002 g folic acid, and 0.001 g vitamin B12. All reagents were dissolved in 1 L H_2_O, sterilized with a 0.22 µm washed filter and aliquoted into 100ml sterile anaerobic bottles. The bottles were purged with N_2_ within the liquid surface for 20 minutes and the headspace for 10 minutes, and then stored at 4°C in the dark.

The 100× trace element stock solution (in one liter) was prepared by dissolving 1.5 g nitriloacetic acid in 800 mL H_2_O, adjusting the pH to 6.5 with KOH, and sequentially adding 0.1 g MnSO_4_·2H_2_O, 0.1 g CoCl_2_·6H_2_O, 0.1 g ZnSO_4_·7H_2_O, 0.01 g CuSO_4_·5H_2_O, 0.025 g NiCl_2_·6H_2_O, 0.2 g Na_2_SeO_4_, 0.1 g Na_2_MoO_4_·2H_2_O, and 0.1 g Na_2_WO_4_·2H_2_O, ensuring each component was fully dissolved before adding the next. The pH was adjusted again to 7.0. The trace element stock solution was sterilized by passing through a 0.22 µm washed filter and aliquoted into 100 mL sterile anaerobic bottles. The bottles were purged with N_2_ in the bottom of the aqueous solution for 20 minutes and the headspace for 10 minutes, and then stored at 4 °C in the dark.

## 1.2 Sample preparation for ultra-thin sections using resin embedding method.

The ultra-thin sections for TEM and NanoSIMS analysis were prepared using the resin embedding method. Cells in the logarithmic growth phase were collected in an anaerobic chamber, centrifuged at 4,000g for 10 minutes, and then fixed in 2.5% glutaraldehyde at 4°C overnight. The next day, the cells were centrifuged at 4,000g for 5 minutes and washed three times with 0.1M phosphate buffer (PB) for 5 minutes. A 1.5% low-melting agarose solution was prepared by dissolving the agarose at 100°C and cooling it to 40°C. The washed cells pellet was mixed immediately with the preheated agarose solution, followed by centrifugation at 10,000g for 1 minute and cooling on ice for 10 minutes. The agarose-embedded cell mixture was then cut into small blocks (~1-2 mm in size) and transferred to glass vials. The embedded agarose blocks were rinsed three times with 0.1M PB buffer for 5 minutes.

The blocks were then stained with 1% osmium tetroxide in the dark for 1 hour, followed by four rinses with water. The blocks were dehydrated using a graded series of ethanol (30%, 50%, 70%, 85%, 95%, and 100%), and then twice with 100% acetone, each for 8 minutes. The embedding resin (Epon812) was prepared by mixing 12.7825g Epon812 resin, 6.755g DDSA (Dodecenylsuccinic Anhydride), and 5.4625g NMA (Nadic Methyl Anhydride), and finally adding 0.5g DMP-30 catalyst (2,4,6-Tris(dimethylaminomethyl)phenol). Samples were infiltrated with a 1:3 (v/v) solution of Epon812 resin and acetone for 1-2 hours, followed by infiltration with a 1:1 (v/v) solution overnight. The samples were then infiltrated with 100% Epon812 resin for 3 hours twice, and a final infiltration was performed overnight. Finally, the samples were transferred to a mold and polymerized at 65°C for 24 hours, and the polymerized sample was used for slicing as required.

## 1.3 RNA extraction, transcriptomic sequencing and data processing and analysis.

Total RNA was extracted using TRIzol reagent (Invitrogen, USA) with slight modifications to the manufacturer's protocol. Cells from 50 mL of culture (equivalent to a total of ~4 × 10^10^ cells) were collected during their logarithmic growth phase by centrifugation at 4,000 g for 15 minutes at 4°C. Three biological replicates were set for each group. The cell pellet was transferred to a Lysis E tube (MP Biomedicals) on ice, and 1 mL of TRIzol reagent was immediately added. The cells were subjected to three cycles of 40 seconds of bead beating followed by 5 minutes of resting at room temperature (~20°C). 200 µL of molecular grade chloroform was added to each tube, mixed by inversion, incubated at room temperature for 3 minutes, and then centrifuged at 12,000 g for 15 minutes at 4°C. The upper aqueous phase containing RNA was carefully transferred to a clean 2 mL tube. To precipitate the RNA, 0.5 mL of pre-chilled (4°C) 100% molecular grade isopropanol was added, and the tubes were incubated on ice for 10 minutes, followed by centrifugation at 12,000 g for 10 minutes at 4°C. The supernatant was discarded, and 1 mL of 75% molecular grade ethanol was added to wash the RNA. After centrifugation at 7,500 g for 5 minutes at 4°C, the supernatant was removed, and the RNA pellet was air-dried for 5-10 minutes. The RNA was dissolved in 50 μL RNA-grade water (Thermo Fisher Scientific, USA) by incubating at 55°C for 10 minutes. Each experiment was performed in three replicates. DNase I (TaKaRa, Japan) was used to remove any DNA impurity according to the instructions. Briefly, the RNA sample was mixed with DNase I and reaction buffer, incubated at 37°C for 20-30 minutes, followed by heating inactivation at 65°C for 8 minutes. After DNase treatment, the RNA was subjected to a second round of isopropanol precipitation, ethanol wash, and resuspension as described above. The RNA concentration was quantified fluorometrically using the Qubit BR RNA kit and Qubit fluorometer (Invitrogen, USA). All samples had concentrations above 50 ng/μL, and the total RNA amount exceeded 2 μg. RNA quality was assessed using a NanoDrop ND-1000 spectrophotometer (Thermo Fisher Scientific, USA) by measuring A260/A280 and A260/230 ratios, with all samples falling within the acceptable ranges of 1.8-2.2 and ≥2, respectively.

High-throughput sequencing was performed using the Illumina platform at Beijing Novogene Technology Co., Ltd. For the sequencing data analysis, Bowtie2 was used to remove residual rRNA sequences. Low-quality reads and adapter sequences were removed using fastp (Chen et al., 2018). The cleaned sequences were then mapped to the Methanococcus maripaludis S2 genome using Salmon for transcript expression quantification and gene annotation (Patro et al., 2017). Quantitative data were analyzed using the R package DESeq2 (Love et al., 2014), identifying genes that were significantly upregulated (log2 fold change>1, padj<0.01) or downregulated (log2 fold change<-1, padj<0.01) under different conditions.

## 1.4 Protein extraction, sample preparation, instrument setup, and data analysis.

Protein extraction and quantification. Cells from 50 mL of culture (equivalent to a total of ~4 × 10^10^ cells) were collected during their logarithmic growth phase by centrifugation at 4,000 g for 15 minutes at 4°C. Three biological replicates were set for each group. To perform proteomics analysis, cells were washed three times with O_2_-free PBS under anaerobic conditions after collection to remove excess salt. Protein extraction was carried out using a lysis buffer containing 8 M urea, 2 M thiourea, 0.1% 3-(3-Cholamidopropyl)-dimethylammonio-1-propanesulfonate, and 1% protease inhibitor cocktail. The cell suspensions were vortexed for 30 minutes and the system underwent three freeze-thaw cycles at -80°C and 65°C for 20 minutes, followed by 30 minutes of vortexing after each cycle. After centrifugation at 15,000 g for 20 minutes and transfer the supernatant to a new 2 mL centrifuge tube and centrifuge for 20 minutes. The protein concentration in the supernatant was measured using a fluorescence-based protein quantification kit (Qubit 4 Fluorometer, QubitTM Protein Assay Kit, Invitrogen, USA). All samples had concentrations above 680 ng/μL, and the total protein amount exceeded 20 μg.

SDS-PAGE and in-gel digestion. For each sample, 20 μg of total protein was separated by SDS-PAGE and stained with Coomassie Brilliant Blue R250 (Macklin, Shanghai). The gel lanes were cut into ~2 mm^3^ cubes, which were then divided into theree fractions for in-gel digestion. In all subsequent washing and digestion steps, sufficient liquid was added to keep the gel pieces fully submerged. The digestion procedure included: washing the fractions with 50 mM ammonium bicarbonate in 40% acetonitrile until the stain faded; washing with 50 mM ammonium bicarbonate and 75% acetonitrile, followed by dehydration with 100% acetonitrile and drying in a vacuum concentrator; reducing with 10 mM dithiothreitol in 25 mM ammonium bicarbonate at 37°C for 2 hours; alkylating free thiol (-SH) groups with 55 mM iodoacetamide in 25 mM ammonium bicarbonate at 37°C for 1 hour in the dark; and washing with 25 mM ammonium bicarbonate and 50% acetonitrile, followed by dehydration with 100% acetonitrile and drying in a vacuum concentrator. The fractions were then rehydrated in 0.01 μg/μL trypsin diluted with 25 mM ammonium bicarbonate and digest for 16 hours at 37°C. The gel block was then incubated with 50% acetonitrile/5% trifluoroacetic acid solution to extract the remaining peptides. This process was repeated twice and the extracts were combined. After vacuum drying, the samples were desalted using RP-C18 StageTip column, and finally dissolved in 10 µL of 0.1% formic acid.

Mass spectrometry analysis. The desalted peptides were analyzed by online nanoscale liquid chromatography (nanoLC, EASY-nLC 1200, Thermo Scientific, USA) coupled with an Orbitrap mass spectrometer (Q Exactive Plus, Thermo Scientific, USA). An injection volume of 1 μL was used for the analysis. The peptides were separated using a two-column system: first, a 2 cm C18 reverse-phase column (inner diameter 75 μm, particle size 3 μm) to remove undigested proteins, followed by a 15 cm C18 reverse-phase column (inner diameter 50 μm, particle size 2 μm) for separation. A two-solvent system was used for separation: Solvent A (3% acetonitrile and 0.1% formic acid) and Solvent B (80% acetonitrile and 0.1% formic acid), with a gradient from 5% B to 95% B (5% A to 95% A) over 120 minutes. Data-dependent acquisition was performed in positive ion mode on the Orbitrap mass spectrometer to collect mass spectra.

Protein identification and quantification. Proteins were identified and quantified with MaxQuant (Version 2.7.5.0) (Tyanova et al., 2016a), filtered using Perseus (Version 2.2.1) (Tyanova et al., 2016b). Quantitative data were analyzed using the R package DESeq2 (Love et al., 2014), identifying proteins that were significantly upregulated (log2 fold change>1, padj<0.01) or downregulated (log2 fold change<-1, padj<0.01).

## 1.5 Data availability

The transcriptomics data are deposited in the National Center for Biotechnology Information (NCBI) under the BioProject accession number PRJNA1193246. The proteomics data are deposited in the iProX platform under project IPX0010456000, subproject IPX00104560001.

# 2 Supplementary Figures and Tables

**Supplementary Figure 1. Comparison of biomass determination methods in the presence and absence of 50 µM FeS_Mack_.** (A) Microscopic image of cells without FeS_Mack_, showing individualcells (indicated by arrows). (B) Microscopic image of cells in the presence of 50 µM FeS_Mack_ showing cell clumping (indicated by arrows). (C and D) Scanning electron microscope images under either Fe^2+^-rich or HS^-^-rich conditions. The sizes of 50 randomly selected cells were measured. The mean cell sizes were 0.805 ± 0.085 μm and 0.768 ± 0.097 μm, respectively. The P value was ≥ 0.05, demonstrating no statistically significant difference in cell size between the two groups. (E) OD_600_ value as a function of cell concentration showing that the presence of 50 µM FeS_Mack_ results in an over-estimate. Data are presented as mean ± standard deviation (n = 3). (F) Total DNA as a function of cell concentration showing that the presence of mineral particles did not interfere with DNA measurement. Data are presented as mean ± standard deviation (n = 3). In most cases, the standard deviations are smaller than the size of the symbol.

**Supplementary Figure 2.** **Methanogenic** **activity and growth of *Methanococcus maripaludis* S2 under different FeS_Mack_ concentrations.** The methanogenic activity and growth were monitored by quantifying changes of total DNA (A, B and C) and methane production (D, E and F) over time. The data represent averages with standard deviations from 3 experimental replicates.

**Supplementary Figure 3. Serial ultrathin-section TEM images of MmS2 cells cultured under the high-Fe^2+^ and high-HS^-^ conditions.** Under the high-Fe^2+^ condition (A, C), electron-dense particles were repeatedly observed within membrane-defined cellular boundaries across adjacent sections, supporting their intracellular localization. Such particles were not observed in cells cultured under the high-HS^-^ condition (B, D) and processed using the same procedure, arguing against a general sample-preparation artifact.

**Supplementary Figure 4. Whole-cell imaging, electron diffraction, and compositional analysis under either Fe^2+^-rich or HS^-^-rich conditions.** Cells were fixed in 2.5% glutaraldehyde and 2% paraformaldehyde, followed by dehydration through a graded ethanol series and final suspension in pure ethanol. Prepared cells were deposited onto carbon-coated copper grids for viewing (Guo et al., 2021). Images were acquired using a field-emission TEM (JEM-F200-SEI). (A) is a secondary-electron (SE) image of the 50 μM FeS_Mack_ + 250 μM Fe^2+^ sample, showing a smooth surface with no particle attachement to the surface, (B) reveals particles in transmission mode, suggesting that they are intracellular. (C) is the corresponding electron diffraction pattern of intracellular particles showing an amorphous nature. A combination of Panel A-C demonstrates that the amorphous particles are located inside the cell. (D) confirms that these are Fe-S particles. (E) does not show any intracellular particles and have no Fe signal on elemental map under the 50 μM FeS_Mack_ + 250 μM HS^-^ condition.

**Supplementary Figure 5. Principal component and pairwise correlation analyses of the transcriptomic and proteomic datasets.** (A, B) Principal component analysis (PCA) of the transcriptomic and proteomic profiles, respectively, under High-HS^-^ and High-Fe^2+^ conditions. Percentages indicate the variance explained by each principal component. (C, D) Pairwise Pearson correlation matrices for the transcriptomic and proteomic profiles, respectively. Values within the matrices represent Pearson correlation coefficients. Each condition included three biological replicates.

**References**

Chen, S., Zhou, Y., Chen, Y., & Gu, J. 2018. fastp: an ultra-fast all-in-one FASTQ preprocessor. *Bioinformatics*, *34*(17), i884-i890. <https://doi.org/10.1093/bioinformatics/bty560>

Love, M. I., Huber, W., & Anders, S. 2014. Moderated estimation of fold change and dispersion for RNA-seq data with DESeq2. *Genome Biol*, *15*(12), 550. <https://doi.org/10.1186/s13059-014-0550-8>

Patro, R., Duggal, G., Love, M. I., Irizarry, R. A., & Kingsford, C. 2017. Salmon provides fast and bias-aware quantification of transcript expression. *Nature Methods*, *14*(4), 417-419. <https://doi.org/10.1038/nmeth.4197>

Tyanova, S., Temu, T., & Cox, J. 2016a. The MaxQuant computational platform for mass spectrometry-based shotgun proteomics. *Nature Protocols*, *11*(12), 2301-2319. <https://doi.org/10.1038/nprot.2016.136>

Tyanova, S., Temu, T., Sinitcyn, P., Carlson, A., Hein, M. Y., Geiger, T., Mann, M., & Cox, J. 2016b. The Perseus computational platform for comprehensive analysis of (prote)omics data. *Nature Methods*, *13*(9), 731-740. <https://doi.org/10.1038/nmeth.3901>
